# Supplementary material for: Oligodendrocytes support functional development of subcortical premotor neurons and navigation
Source: bioRxiv. 2025 Nov 2:2025.11.01.686017. Preprint. [Version 1] doi: 10.1101/2025.11.01.686017 (PMC12636356; doi:10.1101/2025.11.01.686017)
Supplement: Supplement 1 [file NIHPP2025.11.01.686017v1-supplement-1.pdf]

bioRxiv preprint doi: https://doi.org/10.1101/2025.11.01.686017; this version posted November 2, 2025. The copyright holder for this preprint (which was not certified by peer review) is the author/funder, who has granted bioRxiv a license to display the preprint in perpetuity. It is made available under aCC-BY 4.0 International license.

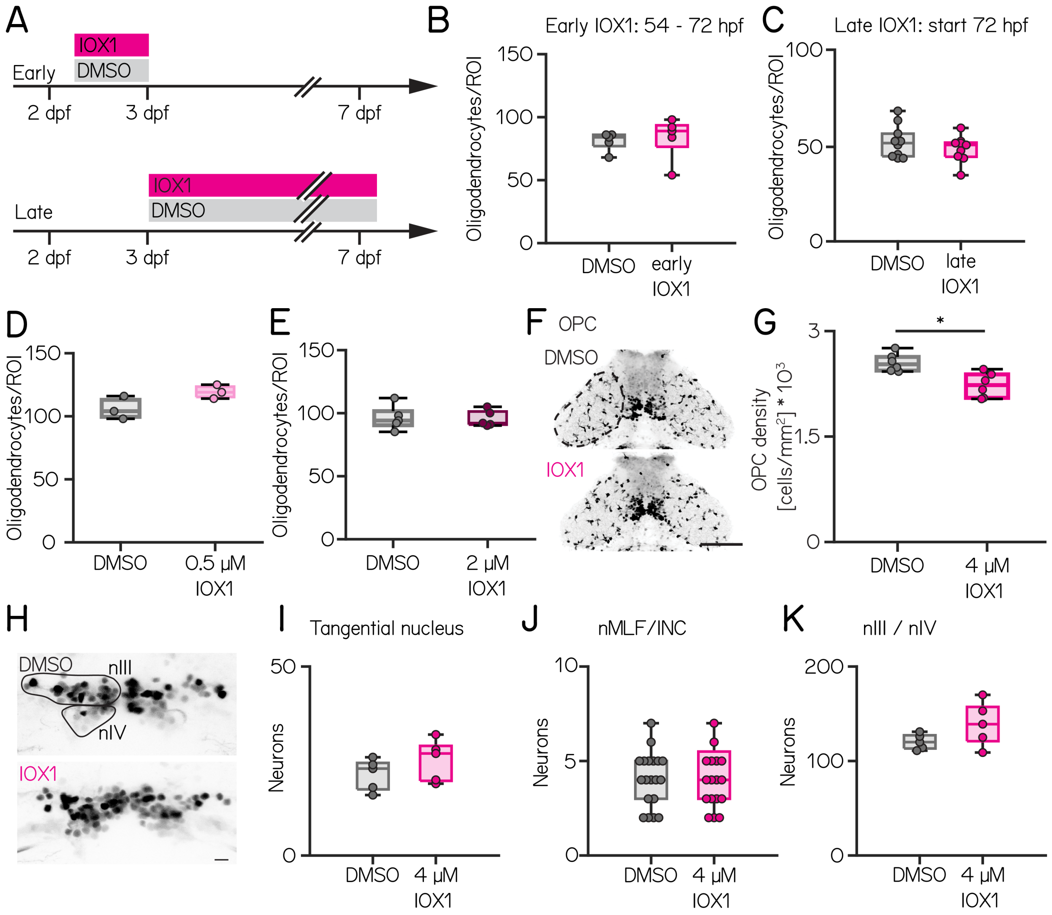

Figure S1: Different timing or concentrations of IOX1 do not affect oligodendrocyte numbers.
(A) Schematic of early (top) and late (bottom) 4 µM IOX1 treatment. White square indicating the analysis timepoint at 7 dpf.
(B) Oligodendrocyte counts at 7 dpf in control (gray) and 4 µM early IOX1-treated larvae (0.81 [0.80–0.82] vs 0.78 [0.77–0.79]; p-value = 0.228).
(C) Oligodendrocyte counts at 7 dpf in control (gray) and 4 µM late IOX1-treated larvae (0.82 [0.81–0.82] vs 0.83 [0.82–0.84]; p-value = 0.485).
(D) Oligodendrocyte counts at 7 dpf in control (gray) and 0.5 µM IOX1-treated larvae (0.77 [0.76–0.78] vs 0.78 [0.77–0.79]; p-value = 0.688).
(E) Oligodendrocyte counts at 7 dpf in control (gray) and 2 µM IOX1-treated larvae (0.76 [0.75–0.77] vs 0.80 [0.79–0.80]; p-value = 0.072).
(F) Confocal image of 7 dpf *Tg(olig2:dsRed)* larvae treated with DMSO (top) or 4 µM IOX1. Dashed region indicating left optic tectum. Scale bar 100 µm.
(G) Oligodendrocyte precursor cell densities in the optic tectum at 7 dpf in control (gray) and 4 µM IOX1-treated *Tg(olig2:dsRed)* larvae (2.5 [2.4 – 2.6] vs. 2.2 [2.0 – 2.4] $*10^3$ cells/mm^2; p-value: 0.0087).
(H) Confocal image of 7 dpf *Tg(isl1a:GFP)* larvae treated with DMSO (top) or 4 µM IOX1. Circled region indicating cranial nuclei nIII and nIV. Scale bar 10 µm.
(I) Tangential nucleus neuron counts at 7 dpf in control (gray) and 4 µM IOX1-treated *Tg(6.7Tru.Hcrtr2:GAL4-VP16)*; *Tg(14xUAS:GCaMP6s)*larvae (DMSO vs IOX1: 23 [18 – 25] vs. 27 [20 – 29]; p-value: 0.2222).
(J) Nucleus of the medial longitudinal fasciculus neuron counts at 7 dpf in control (gray) and 4 µM IOX1-treated *Tg(nefma:hsp70l-LOXP-RFP-LOXP-GAL4)*; *Tg(14xUAS:GCaMP6s)* larvae (DMSO vs IOX1: 4 [3 – 5] vs. 4 [3 – 6]; p-value: 0.8469).
(K) nIII and nIV neuron counts at 7 dpf in control (gray) and 4 µM IOX1-treated *Tg(isl1a:GFP)* larvae (DMSO vs IOX1: 120 [113 – 127] vs. 139 [121 – 157]; p-value: 0.3095).
* indicates p-value < 0.05.

bioRxiv preprint doi: https://doi.org/10.1101/2025.11.01.686017; this version posted November 2, 2025. The copyright holder for this preprint (which was not certified by peer review) is the author/funder, who has granted bioRxiv a license to display the preprint in perpetuity. It is made available under aCC-BY 4.0 International license.

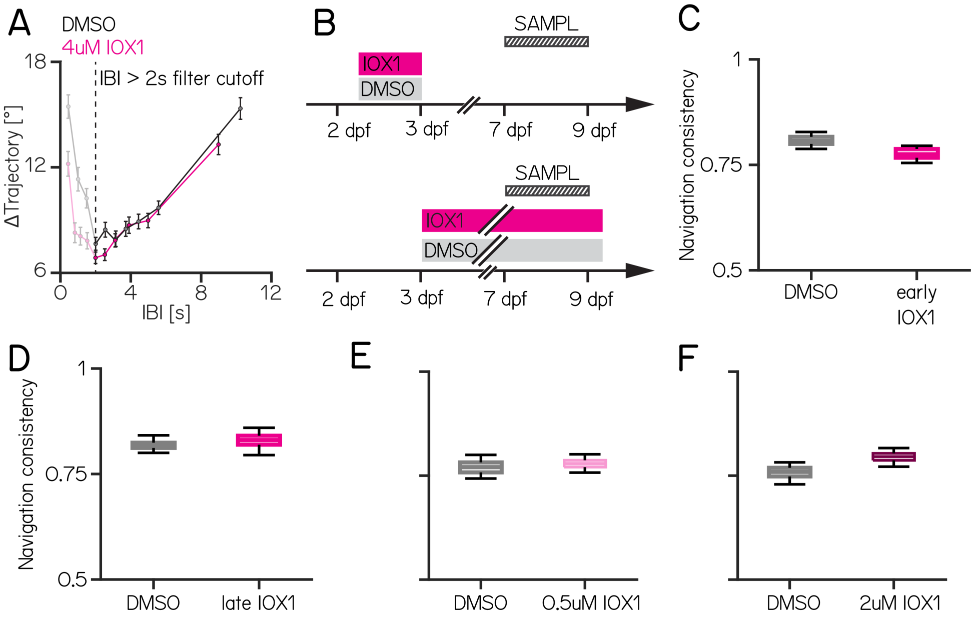

Figure S2: Different timing or concentrations of IOX1 do not affect behavior.
(A) Change in trajectory (delta) of two consecutive bouts as a function of the inter-bout interval (IBI) illustrating the cutoff used for data analysis.
(B) Schematic for early (top) and late (bottom) IOX1 treatment. Striped bars indicate the time of behavior testing.
(C) Navigation consistency for control (gray) and early IOX1 (pink) treated larvae (DMSO vs IOX1: 0.81 [0.80–0.82] vs 0.78 [0.77–0.79] ; p-value: 0.228).
(D) Navigation consistency for control (gray) and late IOX1 (pink) treated larvae (DMSO vs IOX1: 0.82 [0.81–0.82] vs 0.83 [0.82–0.84]; p-value: 0.485).
(E) Navigation consistency for control (gray) and 0.5 μM IOX1 (light pink) treated larvae (DMSO vs IOX1: 0.77 [0.76–0.78] vs 0.78 [0.77–0.79]; p-value: 0.688).
(F) Navigation consistency for control (gray) and 2 μM IOX1 (dark pink) treated larvae (DMSO vs IOX1: 0.76 [0.75–0.77] vs 0.80 [0.79–0.80]; p-value: 0.072).

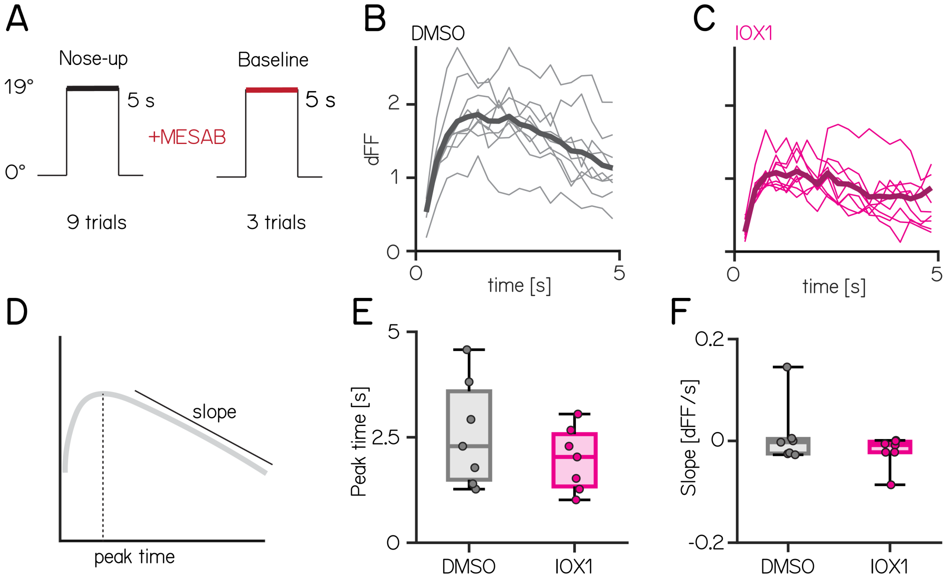

Figure S3: IOX1 treatment does not affect peak fluorescent timing or decay
(A) Stimulus trace for eccentric imaging with anesthetized baseline. Cells are imaged during a 5 s stimulus at 19°. To establish a baseline the same cells are imaged in anesthetized animals.
(B) Example trials of a DMSO treated nMLF/INC neuron,
(C) Example trials of a IOX1-treated nMLF/INC neuron.
(D) Schematic of measured values for E - F.
(E) Quantification of peak time in DMSO versus IOX1-treated animals. (DMSO vs. IOX1: 2.3 [1.5 – 3.6] vs. 2.0 [1.3 – 2.6]; p-value: 0.6591).
(F) Quantification of GCaMP6s slope in DMSO versus IOX1-treated animals. (DMSO vs. IOX1: -0.00007 [-0.0063 – -0.0010] vs. -0.0021 [-0.0057 – -0.0004]; p-value: 0.7104).

bioRxiv preprint doi: https://doi.org/10.1101/2025.11.01.686017; this version posted November 2, 2025. The copyright holder for this preprint (which was not certified by peer review) is the author/funder, who has granted bioRxiv a license to display the preprint in perpetuity. It is made available under aCC-BY 4.0 International license.

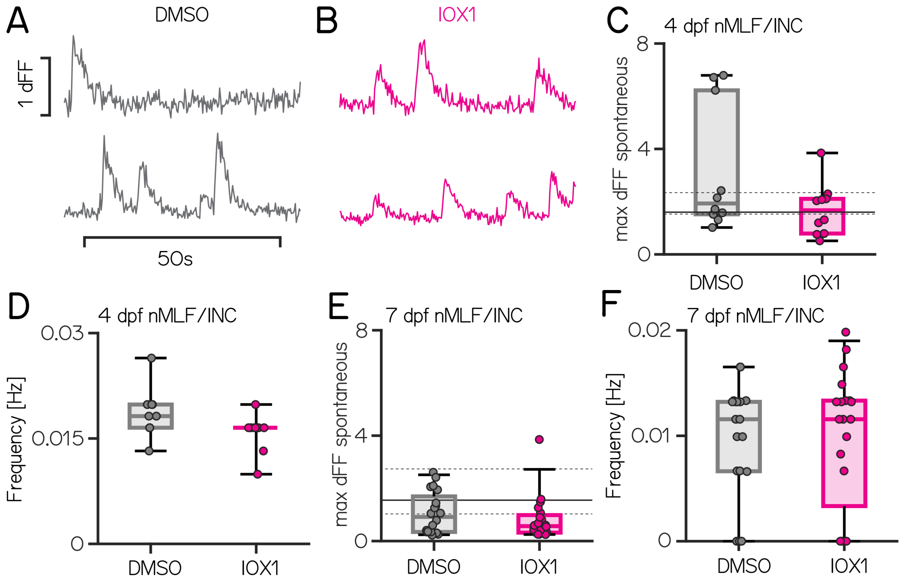

Figure S4: Spontaneous calcium event amplitude or frequency are not affected by IOX1 treatment
(A–B) Example traces of spontaneous calcium events in nMLF/INC neurons at 7 dpf in DMSO (gray) or IOX1 treated animals (pink).
(C) Quantification of spontaneous calcium event amplitude at 4 dpf (DMSO vs. IOX1: 1.93 [1.52 – 6.22] vs 1.67 [0.79 – 2.11]; p-value = 0.162). Full/dashed lines indicate the median and IQR dFF for 4 dpf nMLF/INC nose-up responses.
(D) Quantification of the frequency of spontaneous calcium events at 4 dpf (DMSO vs IOX1: 0.0182 [0.0165 – 0.0198] vs 0.0165 [0.0165 – 0.0165] Hz; p-value: 0.0514)
(E) Quantification of spontaneous calcium event amplitude at 7 dpf (DMSO vs. IOX1: 0.92 [0.35 – 1.7] vs 0.57 [0.32 – 0.98]; p-value = 0.3235). Full/dashed lines indicate the median and IQR dFF for 7 dpf nMLF/INC nose-up responses.
(F) Quantification of the frequency of spontaneous calcium events at 7 dpf (DMSO vs IOX1: 0.0116 [0.0066 – 0.0132] vs 0.0116 [0.0033 – 0.0133] Hz; p-value: 0.7426)

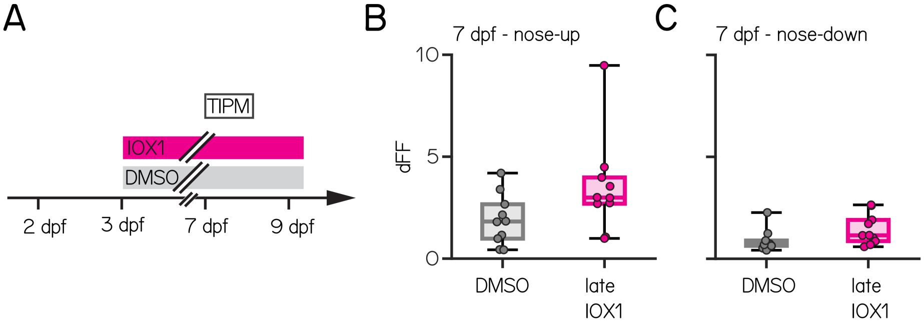

Figure S5: Late IOX1 treatment does not affect nMLF/INC tilt responses
(A) Timeline of the experiment showing IOX1 and DMSO-only treatment timecourse beginning at 72 hours post-fertilization. Imaging timepoint is indicated by the open rectangle at 7 days post-fertilization.
(B) Average responses for 7 dpf nMLF/INC neurons to nose-up stimuli after late IOX1 treatment (DMSO vs late IOX1: 1.82 [0.98 – 2.67] vs. 2.99 [2.69 – 3.98], p-value: 0.0539).
(C) Average responses for 7 dpf nMLF/INC neurons to nose-down stimuli after late IOX1 treatment (DMSO vs late IOX1: 0.71 [0.64 – 0.89] vs. 1.15 [0.87 – 1.91], p-value; 0.0539).

bioRxiv preprint doi: https://doi.org/10.1101/2025.11.01.686017; this version posted November 2, 2025. The copyright holder for this preprint (which was not certified by peer review) is the author/funder, who has granted bioRxiv a license to display the preprint in perpetuity. It is made available under aCC-BY 4.0 International license.

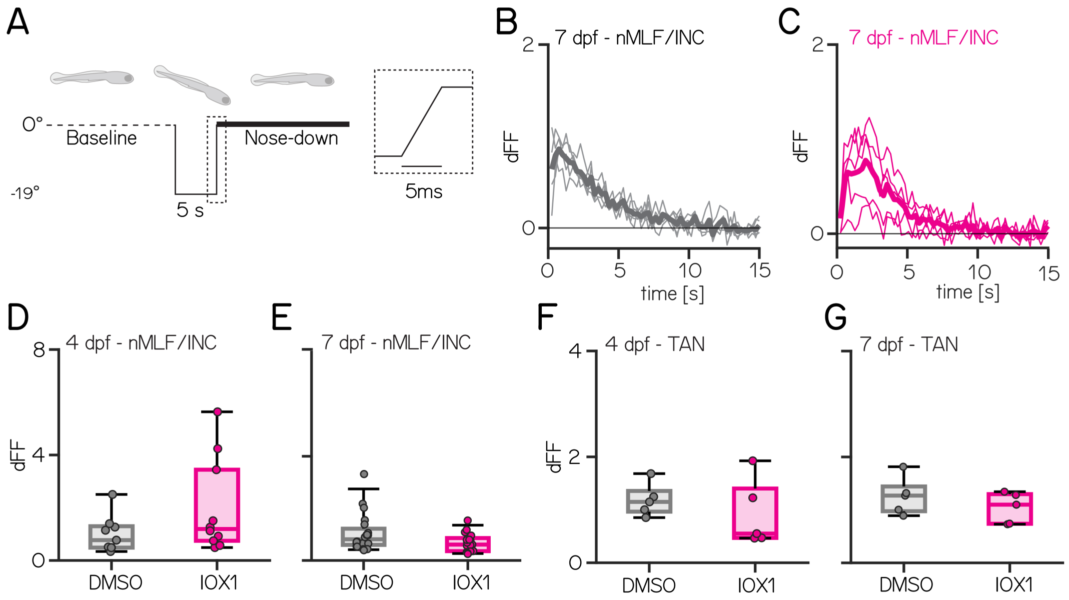

Figure S6: Nose-down pitch tilt responses are not affected by IOX1 treatment

(A) Timecourse of nose-down stimulus for imaging indicating pre-tilt baseline (dotted) and post-tilt imaging (thick line). Dotted rectangle corresponds to the -19° return to baseline.

(B-C) Normalized fluorescent changes after five nose-down tilts in DMSO (E, gray) and IOX1 (F, pink) from 7 dpf fish. Bold line shows the median.

(D) Average responses for 4 dpf nMLF/INC neurons to nose-down stimuli (DMSO vs IOX1: 0.77 [0.49 – 1.30] vs 1.19 [0.75 – 3.44]; p-value: 0.7124).

(E) Average responses for 7 dpf nMLF/INC neurons to nose-down stimuli (DMSO vs IOX1: 0.81 [0.60 – 1.22] vs 0.61 [0.36 – 0.86]; p-value: 0.0869).

(F) Average responses for 4 dpf TAN neurons to nose-down stimuli (DMSO vs IOX1: 1.15 [0.96 – 1.36] vs 0.55 [0.47 – 1.40]; p-value: 0.4206).

(G) Average responses for 7 dpf TAN neurons to nose-down stimuli (DMSO vs IOX1: 1.27 [0.98 – 1.44] vs 1.10 [0.74 – 1.30]; p-value: 0.5476).

bioRxiv preprint doi: https://doi.org/10.1101/2025.11.01.686017; this version posted November 2, 2025. The copyright holder for this preprint (which was not certified by peer review) is the author/funder, who has granted bioRxiv a license to display the preprint in perpetuity. It is made available under aCC-BY 4.0 International license.

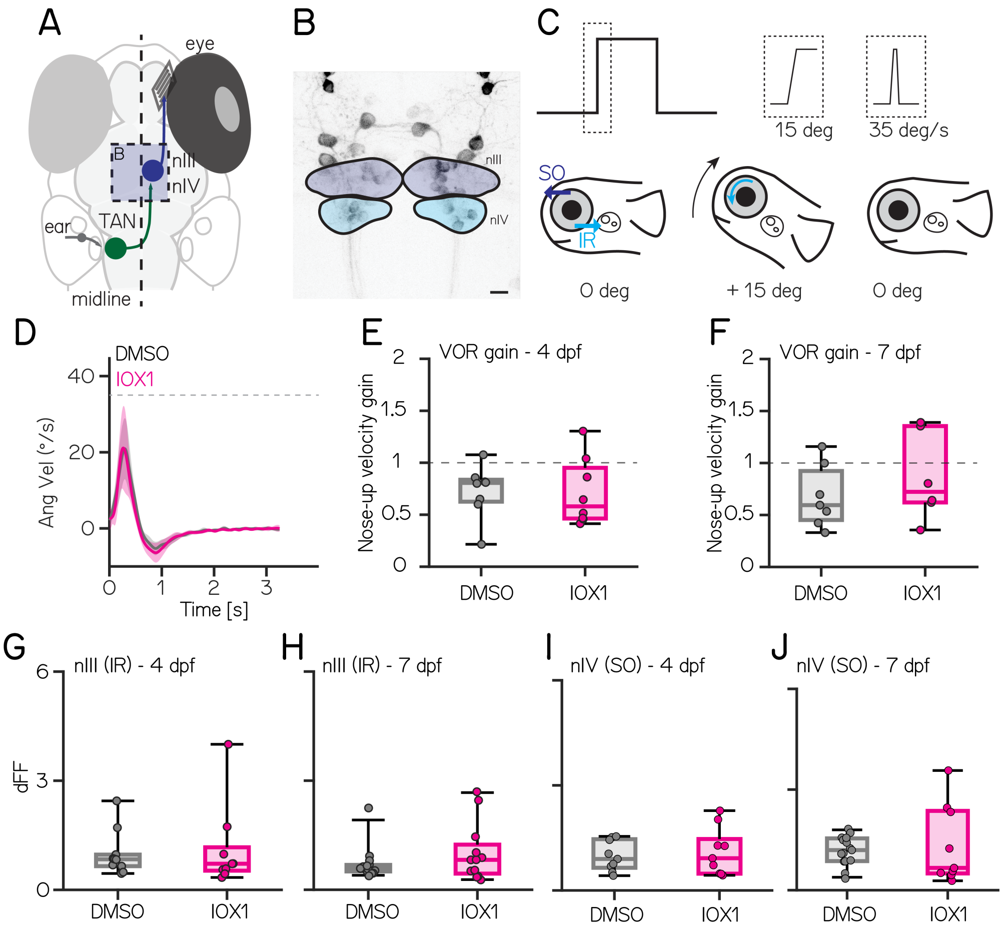

Figure S7: Vestibular ocular reflex circuit and behavior are not affected by IOX1 treatment
(A) Schematic of vestibulo-ocular reflex circuit.
(B) Confocal image of *Tg(nefma:hsp70l-LOXP-RFP-LOXP-GAL4); Tg(UAS:GCaMP6s)* at 7 dpf highlighting the location of nIII (dark blue) and nIV (light blue). Scale bar 10 µm.
(C) Schematic and details of stimulus for eye movement recordings (top). Fish are rotated to 15° nose-up with a maximum velocity of 35°/s. Fish schematic highlighting the location of the two extra-ocular muscles (superior oblique (SO) and inferior rectus (IR)) that rotate the eyes down in response.
(D) Average angular velocity traces of 4 dpf DMSO (gray) and IOX1-treated (pink) animals.
(E) Vestibulo-ocular reflex gain for nose-up stimuli at 4 dpf in DMSO and IOX1-treated larvae (DMSO vs IOX1: 0.81 [0.63 – 0.84] vs 0.58 [0.46 – 0.95]; p-value: 0.7209).
(F) Vestibulo-ocular reflex gain for nose-up stimuli at 7 dpf in DMSO and IOX1-treated larvae (DMSO vs IOX1: 0.59 [0.45 – 0.92] vs 0.72 [0.62 – 1.35]; p-value: 0.3660).
(G) Average responses for 4 dpf nIII neurons to nose-up stimuli (DMSO vs IOX1: 0.84 [0.65 – 0.97] vs 0.72 [0.53 – 1.17]; p-value: 0.8809).
(H) Average responses for 7 dpf nIII neurons to nose-up stimuli (DMSO vs IOX1: 0.59 [0.50 – 0.68] vs 0.82 [0.44 – 1.2]; p-value: 0.4809).
(G) Average responses for 4 dpf nIV neurons to nose-up stimuli (DMSO vs IOX1: 0.90 [0.65 – 1.46] vs 0.92 [0.48 – 1.47]; p-value: 1.0000).
(H) Average responses for 7 dpf nIV neurons to nose-up stimuli (DMSO vs IOX1: 1.20 [0.87 – 1.54] vs 0.66 [0.50 – 2.36]; p-value: 0.6764).
